# Promoting public access to clinical trial protocols: challenges and recommendations

**DOI:** 10.1186/s13063-018-2510-1

**Published:** 2018-02-17

**Authors:** An-Wen Chan, Asbjørn Hróbjartsson

**Affiliations:** 1 Women’s College Research Institute, Women’s College Hospital, 76 Grenville St, Rm 6416, Toronto, ON M5S 1B2 Canada; 2 Department of Medicine, University of Toronto, Toronto, ON Canada; 3 Center for Evidence-Based Medicine, Odense University Hospital, Odense, Denmark; 4 Department of Clinical Research, University of Southern Denmark, Odense, Denmark

**Keywords:** Clinical trial protocol, Publication, Transparency, Selective reporting

## Abstract

**Background:**

Recognizing the value of promoting public access to clinical trial protocols, *Trials* pioneered the way for their publication over a decade ago. However, despite major advances in the public accessibility of information about trial methods and results, protocol sharing remains relatively rare.

**Main body:**

Protocol sharing facilitates the critical appraisal of clinical trials and helps to identify and deter the selective reporting of outcomes and analyses. Challenges to the routine availability of high quality trial protocols include the gaps in incentives and adherence mechanisms, limited venues for sharing the original and final protocol versions, and the need for mechanisms to ensure transparent and complete protocol content.

**Conclusions:**

We propose recommendations for addressing key challenges to protocol sharing in order to promote routine public access to protocols for the benefit of patients and other users of evidence from clinical trials.

## Introduction

As the cornerstone of medical evidence, clinical trials have been instrumental to major healthcare advances over the past decades. However, the impact and value of clinical trials have been limited by inaccessible or selectively reported information about their methods and results, leading to substantial research waste with direct implications for healthcare costs and patient outcomes [1].

Public access to study protocols is fundamental to the societal value of clinical trials. The trial protocol is the core document reporting the study background, relevance, methods, administration, and ethical considerations [2]. The protocol guides the study team to help ensure that the trial is implemented in a manner that is consistent with the research objectives and the intentions of the steering group. Prior to recruiting study participants, the protocol serves as the basis for trial registration and for external review by research ethics committees and regulators. Increasingly, the protocol is also reviewed after trial completion to place its results into proper context.

In 2006, *Trials* pioneered the way for public access to trial protocols by providing a venue for their publication [3]. Over 10 years later, the value of public access has become generally accepted by trialists, sponsors, funders, journal editors, regulators, healthcare providers, and patients. This acceptance has manifested in the growing number of protocols made available in journals as stand-alone publications or as web supplements accompanying published final reports [4]. In *Trials* alone, the number of published protocols has increased from 46 in 2008 to 167 in 2012 and 359 in 2016.

However, despite repeated calls for increased protocol sharing [5–7], most clinical trials do not have a publicly accessible protocol [8–10]. In this commentary we review the rationale and challenges, and propose recommendations for improving public access to protocols.

## Benefits of protocol sharing

Public availability of study protocols helps to facilitate detailed assessments of the internal validity of a trial, deter selective reporting of outcomes and analyses, and improve understanding of external validity. This key role is becoming even more relevant with the introduction of policies requiring the registration of summary results on trial registries as well as the expanded sharing of participant-level data [11–13].

### Internal validity

The protocol contains information that is essential to appraising internal validity (i.e., risk of bias). It is otherwise difficult to evaluate and interpret the results of a trial without access to sufficient information on its design, conduct, and analysis. Published final reports often lack adequate descriptions of important design elements such as the methods of randomization and blinding [14–18]. Combined with the lack of publicly available protocols, these deficiencies in published final reports contribute to the finding that 89% of randomized trials included in systematic reviews had an ‘unclear’ rating for at least one domain of the Cochrane risk of bias tool, which is widely used for evaluating the internal validity of trials [19]. In contrast, a high-quality protocol is not subject to word count restrictions and provides a comprehensive view of the pre-specified study methods.

### Selective reporting

Access to a greater level of detail in the protocol also provides a key mechanism to identify and deter the selective reporting of outcomes and analyses. Unacknowledged discrepancies in the primary outcomes, sample size calculations, and analysis plans are often found in published final reports when compared with the protocol [20–25]. Selective reporting within published reports acts in the same direction as publication bias of whole trials. Collectively, these reporting biases tend to inflate the efficacy estimates of individual trials and meta-analyses.

The protocol can also help to clarify important aspects of trial organization related to the roles of individuals, sponsors, and funders in trial design, conduct, and reporting. Protocols provide information on who controls or owns the trial data, who makes the decision for early trial stoppage, and the relationship between protocol contributors, professional medical writers, and authors of the final study report [26–29]. Protocols will also often describe any restrictions to the publication of trial results [26]. This type of information is essential for adequate assessment of the risk of bias.

### External validity

Availability of the protocol facilitates an understanding of the external validity (i.e., generalizability) of trial results. To determine how the findings should be applied in practice to individual patients, it is necessary to evaluate whether a given clinical scenario differs substantively from how the trial was conducted. Relevant considerations include the study setting, selection of trial participants and centers, details of the interventions and their administration, follow-up intensity, and concomitant care [30]. Many of these trial elements are inadequately described in published final reports.

The protocol, in contrast, conveys much more information about these clinically relevant elements. For example, the eligibility criteria defined in the protocol are often not fully described in the published final report, which tends to portray a broader eligible study population than the protocol [31–33]. Further, the trial interventions are more completely described in protocols than in the published final reports, which is relevant when applying the interventions in clinical practice [34].

### Complement to trial registries and results databases

While major progress in trial registration has been made over the past decade, the adherence levels and quality of registered information are highly variable [22, 35–41]. The Trial Registration Data Set defined by the World Health Organization and endorsed by the International Committee of Medical Journal Editors provides a brief outline of a trial’s topic and design [42]. However, the methodological information in registries is usually insufficient to appraise the merits of the study design or identify selective reporting of analyses [43]. For example, a recent study of registered and published oncology trials found that, due to incompletely or inaccurately registered information, the registry record enabled detection of only 75% of cases of discrepant primary outcomes in the published final report compared with using the full protocol [44]. These limitations of trial registration highlight the important complementary role of having access to full protocols.

## Challenges and recommendations

Despite its important benefits, public access to protocols is not yet widespread. Challenges to the routine availability of high quality trial protocols include the gaps in incentives and adherence mechanisms, limited venues for sharing the original and final protocol versions, and the need for mechanisms to ensure transparent reporting and completeness of protocol content. We propose recommendations to help address each of these challenges (Table 1).

**Table 1:** Challenges and recommendations for promoting access to full trial protocols

Challenge	Recommendation
Adequate incentives	Academic institutions and funders should implement research assessment indicators that give explicit credit to investigators who share protocols for their ongoing and completed trials
Comprehensive adherence mechanisms	Journal editors, regulators, sponsors, and funders should implement and enforce policies requiring public sharing of protocols for all trials within their remit
Prospective access to the original protocol	The original protocol version receiving ethics approval should be shared or placed in a lockbox prior to participant enrolment, to be made available at the time of results reporting along with the final protocol version listing any amendments
Universal venue for sharing protocols	Trial registries and journals should build capacity to become the standard repositories for housing and publishing of the original and final protocols online
Complete protocol content	Trial protocols should address key elements defined in the SPIRIT (Standard Protocol Items: Recommendations for Interventional Trials) guidance [2, 67]

### Adequate incentives

In the absence of a universal adherence mechanism, it is important to provide sufficient incentives for trial investigators to share protocols as part of a broader dissemination plan that includes the full reporting of study results and participant-level data [1]. Protocol publication provides credit in the form of a citable paper. Having a protocol that is open to public vetting also provides a degree of transparency that can benefit the trial and its investigators by boosting public awareness and the perceived trustworthiness of a trial.

Public sharing of protocols should be explicitly recognized by academic institutions and funders as a meritorious component of research performance evaluations. Rewarding the dissemination of full trial information helps to recognize its value in reducing research waste and increasing the impact of a study. There is a growing movement towards evaluating researchers based on comprehensive qualitative and quantitative indicators of impact rather than solely relying on traditional bibliometric and funding indices [45–48].

A clear disincentive for protocol sharing is when investigators have signed agreements with sponsors or funders that inappropriately restrict their freedom to disseminate the protocol or other essential information related to the trial [26, 49–51]. It is critical for sponsors, funders, and investigators to avoid placing such restrictions when agreeing to collaborate on a trial.

### Comprehensive adherence mechanisms

Journal editors, regulators, sponsors, and funders can play a vital role by implementing policies mandating protocol availability [1]. Because each stakeholder has a limited scope of trials within its purview, there is a need for broad participation across and within all stakeholder groups. Requiring the submission of protocols to journals along with manuscripts was suggested (and rejected) as far back as 1990 [52]. Although most journals still do not routinely publish trial protocols, some major journals, such as *The BMJ*, *Lancet*, *PLoS Medicine*, and *Annals of Internal Medicine,* have taken the lead by requiring the submission of protocols and posting them online alongside trial manuscripts [53–56].

Regulatory authorities also have a critical role. In 2015, after Gøtzsche and Jørgensen’s pioneering efforts to obtain access [57], the European Medicines Agency implemented a policy to grant online access to clinical study reports (including protocols) that it reviewed as part of marketing authorization submissions [58]. In the United States, a recent clarification of the Food and Drug Administration Amendments Act of 2007 affirmed the vital role of protocols and required that they be submitted at the time of registration of summary results on ClinicalTrials.gov [12]. Although these major advances have not yet had sufficient time to demonstrate their impact, a substantial limitation is that almost half of non-industry sponsored trials fall outside of the scope of legislative requirements [59]. Legislation in the United States and European Union excludes phase 1 trials or those evaluating interventions other than regulated drugs and devices [12, 58].

Additional key stakeholder groups include industry and non-industry sponsors and funders. In 2013, the European Federation of Pharmaceutical Industries and Associations endorsed the voluntary sharing of trial protocols, results, and participant-level data for research purposes [60]. An online industry portal has been established to provide access to this information for researchers, subject to approval of the specific request [61]. However, there is substantial variability across participating companies in terms of the scope of disclosure policies as well as the adherence to them [62, 63]. Some companies, such as GlaxoSmithKline, have gone further by voluntarily posting a subset of their protocols on their own publicly accessible website [64].

Since the patchwork of existing editorial, regulatory, and sponsor policies capture only a fraction of trials conducted around the world, it is important that journal editors, regulators, sponsors, and funders implement measures to improve adherence on a broader scale. Following the lead of major journals, all journals that publish a clinical trial report should either post the protocol as a web supplement or post a link to the online protocol publication. Regulatory policies mandating public access to protocols should be adopted by governments as a condition of marketing authorization. Both industry and non-industry sponsors should make their protocols available for all trials under their stewardship, while funders should require protocol sharing as a condition for grant approval.

### Prospective access to the original protocol

Current journal policies and legislation call for protocol sharing prior to the completion of data collection or at the time of results reporting or regulatory submission [12, 58, 65]. This delayed timing allows the final protocol version to be shared, including a list of any amendments, since protocols often evolve with several formal versions over the course of a trial [66].

However, a major concern is the potential for biased, undisclosed amendments to the pre-specified trial outcomes or analyses based on interim examination of the data. While it is expected that amendments would be transparently listed in each protocol version [67], we and others have found that even revisions to the protocol-defined primary outcomes are common and not acknowledged in the published final reports or latest protocol version [20, 22–24, 68, 69].

Public access to both the original protocol version dated prior to participant enrolment, as well as the final protocol version with a list of amendments, would provide a verifiable record to help identify and evaluate any unacknowledged amendments. Although concerns have been raised over competitive advantage associated with earlier disclosure of detailed protocol information [70–73], the European Medicines Agency and legal rulings in the United States have concluded that very little of the information contained in a trial protocol constitutes commercially confidential information that would confer a competitive advantage if disclosed to other sponsors [74–76]. Earlier protocol sharing helps to assert the intellectual origin of the trial idea. The originators would have a substantial head start with funding, ethics approval, and logistical implementation of the trial. Further, the basic trial description should already be publicly documented on a trial registry prior to participant enrolment. The widespread adoption of trial registration policies reflects broad acceptance that the benefits of public access outweigh the potential risks.

In rare cases where legitimate concerns remain about prospectively sharing the full protocol prior to trial inception, a potential solution would be to offer a lockbox whereby the original protocol receiving ethics approval is submitted to a registry or journal prior to participant enrolment, but not made publicly available until a later time prior to data unblinding. For example, in the context of certain trials evaluating complex interventions where participants are blinded to the true nature of the placebo arm or study hypotheses, a lockbox can help to address concerns about unblinding when participants have access to the protocol while the trial is ongoing [77].

### Universal venue for sharing protocols

Trial registries already provide an established, unrestricted, searchable online mechanism for recording basic protocol information for a trial. A natural extension of this important role would be for registries to serve as the standard repository for housing the original and final trial protocols alongside each registry record. ClinicalTrials.gov and the Australian New Zealand Clinical Trials Registry have recently allowed the full protocol and related documents to be uploaded [12, 78]. To ensure that protocols are captured for all trials regardless of where they are registered, it is important to build protocol uploading capacity for all registries in the World Health Organization Registry Network.

Another key venue for protocol dissemination is journals. Protocol publication offers citable indexing in Medline or other bibliographic databases. There is also the opportunity for additional explanation and discussion of topical issues, beyond the protocol text. However, it is usually not possible to implement major design modifications in response to peer reviewer comments, given that the protocol has already been approved by a research ethics committee, funder, or regulator. Another limitation is that the protocol published in journals is often an abbreviated version of the original protocol, which can reduce transparency if the published version omits relevant details about the trial. Further, given the small number of journals that currently publish protocols, there would be limited capacity to efficiently handle the potential volume of protocol submissions for the thousands of trials initiated monthly [79].

As protocol publication becomes more widespread, it will be important for journals to commit the necessary resources to review and make decisions on submissions in a timely manner. Journals that publish abridged versions of trial protocols should include the full protocol as an online appendix (for example, see [80]), similar to how some journals post the full protocol alongside the final report of trial results. After trial completion, the final protocol version could also be posted as a supplementary appendix to the original published protocol.

### Complete protocol content

The sharing of a protocol is only useful if the document adequately details the key features of a trial. Protocols are usually more informative about study design and organization than published trial reports, but many protocols still lack information about important methodological, ethical, and administrative trial elements [2]. Missing or unclear information in the protocol makes it difficult to know whether or not the issues were adequately handled by the trial investigators. Poor quality protocols also lead to increased time and costs required for trial completion [66].

To help promote high-quality and complete content, protocols should adhere to the SPIRIT (Standard Protocol Items: Recommendations for Interventional Trials) 2013 Statement and explanatory paper, which define a minimum checklist of items to address [2, 67, 81]. SPIRIT has been endorsed internationally by over 100 medical journals, including *Trials* [65], as well as research organizations and funders. An online, SPIRIT-based protocol authoring tool is being developed to make it easier to create, manage, and register high-quality protocols [82].

## Conclusions

Ensuring public access to protocols adds considerable value and reduces research waste by providing important study details that are often not found in other sources of information. The routine sharing of protocols with pre-defined outcomes and analysis plans is a simple, low cost, and feasible way to deter and identify selective reporting. However, key challenges must be addressed to fully realize the positive impact of protocol availability. With ongoing and expanded support from journal editors, trial registries, sponsors, funders, regulators, and legislators, public access to study protocols can become standard practice for the benefit of patients and other users of evidence from clinical trials.

## Abbreviations

SPIRIT: Standard Protocol Items: Recommendations for Interventional Trials
